# Supplementary material for: Identification of photoperiod-induced specific miRNAs in the adrenal glands of Sunite sheep (Ovis aries)
Source: Front Vet Sci. 2022 Jul 22;9:888207. doi: 10.3389/fvets.2022.888207 (PMC9354845; doi:10.3389/fvets.2022.888207)
Supplement: Supplementary file 1 [file Data_Sheet_1.ZIP › Supplementary file/miRDeep2 file.zip!/miRDeep2 file/Novel_Structure/SP42A1/Novel_275.pdf]

Provisional ID	:	NC_019467.2_13259
Score total	:	2.5
Score for star read(s)	:	-1.3
Score for read counts	:	0
Score for mfe	:	2.2
Score for randfold	:	1.6
Score for cons. seed	:	
Total read count	:	72
Mature read count	:	70
Loop read count	:	0
Star read count	:	2

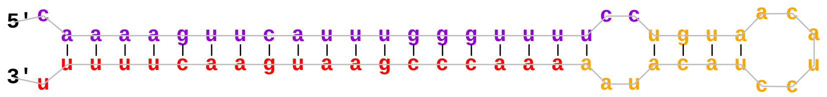

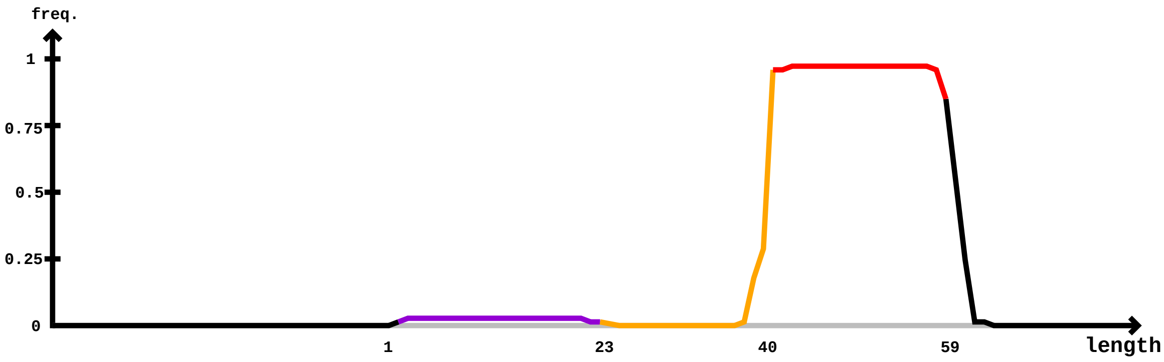

Star Mature

```
5'- acacuaggagacaccuagguucuauuggguuggccaaaaguucauuuggguuuuccuguaacauccuacauaaaaacccgaaugaacuuuuuagccaacucaauacauacg   -3'   obs
    acacuaggagacaccuagguucuauuggguuggccaaaaguucauuuggguuuuccuguaacauccuacauaaaaacccgaaugaacuuuuuagccaacucaauacauacg         exp
    ...(((((.....)))))....((((((((((((.((((((((((((((((((..((((......))))..))))))))))))))))))..))))))))))))......    reads   mm   sample
    ..................................caaaaguucGuuuggguuuucc.......................................................    1       1    seq
    ...................................aaaGguucauuuggguuuu.........................................................    1       1    seq
    ......................................................................uaaaaacccgaaugaacuuuu....................    1       0    seq
    .......................................................................aaaaacccgaaugaacuuu.....................    1       0    seq
    .......................................................................aaaaacccAaaugaacuuuu....................    2       1    seq
    .......................................................................aaaaaccAgaaugaacuuuuu...................    2       1    seq
    .......................................................................aaaaacccgaaugaacuuuuGa..................    1       1    seq
    .......................................................................aaaaacccAaaugaacuuuuua..................    4       1    seq
    .......................................................................Gaaaacccgaaugaacuuuuua..................    2       1    seq
    ........................................................................aaaaccAgaaugaacuuuu....................    2       1    seq
    ........................................................................aaaaccAgaaugaacuuuuu...................    4       1    seq
    ........................................................................aaaacccAaaugaacuuuuua..................    2       1    seq
    .........................................................................aaacccgaaugaacuuuu....................    3       0    seq
    .........................................................................aaacccgaaugaacuuuuC...................    2       1    seq
    .........................................................................aaacccgaaugaacuuuuu...................    19      0    seq
    .........................................................................aaacUcgaaugaacuuuuu...................    1       1    seq
    .........................................................................aaacccgaaugaacuuuuua..................    5       0    seq
    .........................................................................aaacccgaaugaacuuuuuC..................    1       1    seq
    .........................................................................aaacccAaaugaacuuuuua..................    1       1    seq
    .........................................................................aaacccgaaugaacuuuuuGg.................    17      1    seq
    ...........................................................................acccgaaugaacuuuuuGgcc...............    1       1    seq
```
